# Bioelectrical impedance vector analysis and body composition in cervical spinal cord injury: A pilot study

**DOI:** 10.3389/fnut.2022.935128

**Published:** 2022-08-01

**Authors:** Andreia Bauermann, Anselmo de Athayde Costa e Silva, Flávia Figueiredo, Josely Correa Koury

**Affiliations:** ^1^Graduate Program in Human Movement Sciences, Federal University of Pará, Belém, Pará, Brazil; ^2^Brazilian Paralympic Academy, São Paulo, Brazil; ^3^Brazilian Paralympic Committee, São Paulo, Brazil; ^4^Nutrition Institute, Rio de Janeiro State University, Rio de Janeiro, Brazil

**Keywords:** bioelectrical impedance, body composition, spinal cord injuries, tetraplegia, wheelchair sports

## Abstract

**Introduction:**

Body composition assessment in cervical spinal cord injury (c-SCI) individuals is important to monitor the fat free-mass (FFM) loss, due to immobilization, or gain, due to exercise practice. Single frequency bioelectrical impedance analysis (SF-BIA) is low in cost, simple and easy.

**Objectives:**

The aims of this study are: to evaluate the concordance between the FFM values obtained using dual X-ray absorptiometry (DXA) and the three SF-BIA previous predictive equations; and to test the applicability of the bioelectrical impedance vector analysis (BIVA).

**Methods:**

Twenty-three c-SCI males were divided into two groups: Physically active (PA; *n* = 13; at least 150 min/week) and non-active individuals (NPA) and were assessed by DXA and SF-BIA simultaneously.

**Results:**

FFM values were similar between groups PA and NPA. Considering all participants, FFM values obtained by Kocina and Heyward (>11%) and Sun (<15.4%) predictive equations were different when compared to DXA (*p* < 0.01). However, Buchholz's et al. predictive equation showed FFM values similar to DXA, but presented poor concordance (<7%, *p* = 0.99; concordance coefficient = 0.85). BIVA showed consistency in ellipse distribution using FFM obtained using Buchholz et al. predictive equation.

**Conclusions:**

The use of non-specific BIA equations can lead to misinterpretation in FFM values in male c-SCI individuals. Predictive equations for this group need to be developed.

## Introduction

Cervical spinal cord injury (c-SCI) implies serious complications, principally skeletal muscle atrophy, which are associated with adverse metabolic effects, including glucose intolerance, insulin resistance, type II diabetes and cardiovascular disease (1–3). Regardless of the age group, as time advances, SCI individuals present a body composition profile similar to that of the elderly, with reduced fat-free mass (FFM) and increased fat mass due to reduced physical capacity.

The practice of physical exercises in c-SCI can have beneficial consequences on body composition and metabolic changes (4–6). Exercise practice has been considered an important strategy to improve FFM and reduce fat mass, decreasing risks of developing cardiometabolic diseases in different population groups (7–9). FFM is an important component of body composition, and it is related to the development of strength, power, physical performance (10, 11).

FFM can be assessed by high-accuracy methods, such as dual X-ray absorptiometry (DXA) (12, 13). However, DXA is not portable and is highly costly. On the other hand, methods such as bioelectric impedance analysis (BIA) are more affordable and portable (14). BIA is non-invasive, simple, accurate and relatively inexpensive, and it seems to be a promising method for assessing body composition in SCI individuals (13, 15–17).

BIA method estimate FFM from body electrical proprieties, the resistance (R) and reactance (Xc) vectors are used in predictive equations which consider sex, age, height and weight (18). Kocina and Heyward FFM predictive equation is the only BIA predictive equation validated for SCI individuals compared to FFM obtained using DXA (19). Buchholz et al. and Desport et al. validated predictive equations for SCI individuals, and the results were compared to total body water (TBW) obtained using the deuterium method. The equation developed by Buchholz et al. was aimed for chronic SCI individuals, and the one developed by Desport et al. was aimed for healthy elderly individuals. Panisset et al. and Desneves et al. observed the BIA applicability of FFM prediction in acute SCI individuals (20, 21). Both studies used a derivative of BIA known as bioimpedance spectroscopy (BIS), which measures impedance over a range of frequencies, allows the current to pass through and around the cell membrane, and provides more precise measurements of extracellular and intracellular fluid volume (22).

The spinal cord contributes to venous return through muscle contractions. In high SCI, the lack of muscle contractions can cause edema, mainly in the lower limbs (23). This characteristic makes it difficult to assess FFM using the BIA method in SCI individuals, since the BIA method assumed values for body density of 1.05 g/mL and for hydration fraction was 0.732 (24).

Bioelectrical impedance vector analysis (BIVA) is recommended for further nutritional assessment and monitoring, in particular when calculation of body composition is not feasible (25, 26), as in SCI individuals. BIVA is the graphical representation of direct measurements of the z-score of the R and Xc vectors (z-score R-Xc graph), and are plotted as bivariate vectors with tolerance intervals in the R-Xc plane (27). BIVA reflects differences in the bioelectric patterns and permits monitoring of the evolution of the nutritional status and changes associated with body composition, when comparing individual vectors and ellipses to reference populations (28, 29).

Therefore, the aims of this study were: (a) to evaluate the concordance between the FFM values obtained using dual X-ray absorptiometry (DXA) as a reference method and the three SF-BIA previous predictive equations related to SCI, and one predictive equation related to elderly people; and (b) to test the applicability of BIVA in physically active and non-physically active chronic c-SCI individuals.

## Materials and methods

### Participants

Twenty-three c-SCI individuals volunteers with a lesion between C5 and C7 participated in the present study. They were divided into two groups according to the frequency of physical exercise: Physically active which were wheelchair rugby players (PA; *n* = 13; age = 25.0 years (IQR 23.0–33.0); had practiced physical exercise for at least 3 months, 3 times/week or more, totaling a minimum time of 150 min/week) (30); and non-physically active [NPA; *n* = 10; age = 36.5 years (32.8–40.0)]. General information was obtained through a structured questionnaire; the participants answered questions about time of injury, and how long they practiced physical sports. None of the participants were taking nutritional supplements. All participants voluntarily agreed to participate in the research, and written informed consent was obtained.

Although the sample is small, it is important to emphasize that all subjects are quadriplegic with lesions between c5 and c7, which makes the group homogeneous. Furthermore, the sample obtained here is consistent with the literature (31) showing that in investigations with subjects with SCI, the sample size is generally small. This study was approved by the National Research Ethics System (COEP 052/2009). The individuals were instructed not to eat for 4 h and to drink enough water to maintain hydration. All measurements were taken in a single day.

### Anthropometric measurements and body composition

Length was measured from the top of the head to the bottom of the heel using a stadiometer (Seca^®^). The stadiometer was extended on the stretcher of the DXA, where the individuals were placed in the supine position.

Total body mass, total fat, and fat-free mass were determined by Lunar iDXA device with enCore 2008 software version 12.20 (GE Healthcare, Madison, Wisconsin, USA). The participants were placed in a dorsal position. Orthopedic surgical pins or other implants were identified as artifacts, and the software did not include them in the analysis. The exams were performed by a single trained and qualified professional, following the quality control procedures recommended by the manufacturer and the official recommendations of the International Society for Clinical Densitometry (32). Measurements on the calibration block (daily) and on the calibration spine phantom (weekly) supplied by the manufacturer had coefficients of variation 0.7%. Soft tissue body composition, i.e., fat mass (kg), body fat mass (percentage), and lean tissue mass (kg), were derived from the total body scan. Total body mass (kg) was taken as the sum of fat mass, lean tissue mass and bone mineral content.

Body composition (FFM and FM, kg) was assessed using a single frequency (SF) bioimpedance analyzer (RJL, 101 Quantum, Detroit, MI), applying foot-to-hand technology. In order to avoid clinical disturbances in fluid distribution, participants were instructed to abstain from food and liquids for 4 h and to abstain from caffeine and physical activity for 24 h before SF-BIA. Bladder emptying was performed by catheter valve before arrival at the laboratory. Before each test, the analyzer was checked with the impedance calibration (resistance *R* = 500 ohms); and the components inside the bioimpedance analyzer, such as the signal generator, the sensing apparatus, the scales of weight and height, and the electrical interference were tested as suggested by Kyle et al. The average of the two repeated measurements of R and Xc was used in the subsequent analyses. SF-BIA was performed for FFM prediction, two electrodes placed on the dorsal surface of the hands and two on the surface of the feet. The device was then connected, and the voltage was detected by proximal electrodes, and the values of R and reactance (Xc) were obtained.

The predictive equations were selected based on the following criteria: (1) validated for SCI individuals or elderly, ideally using DXA; (2) BIA analyzer used to develop the equation at 50 kHz; (3) sex as a variable of the predictive equation. Three proposed predictive equations met the inclusion criteria: Kocina and Heyward (19), validated BIA predictive equation to estimate the FFM considering 91 adults with spinal cord injury and used DXA as a reference value; Buchholz et al. (33) FFM predictive equation, developed to elderly and validated to paraplegic individuals; and Sun et al. (34) predictive equation, validated using FFM obtained using DXA, developed with representative able-bodied adults, which include elderly people, and recommended for epidemiological studies. Table 1 shows the details of the selected equations.

**Table 1:** BIA equations found for the prediction of FFM in SCI individuals and able-bodied people^*^.

**Author**	***n* (sex)**	**Age (years)**	**Characteristics**	**Predictive equations**	**SEE (kg)**	***R* ^2^**
Sun et al. (34)*	1,829 (734 males)	12–94	Abled-bodied	FFM = −10.68 + 0.65 Ht^2^/R + 0.26 W + 0.02 R	0.4	0.90
Kocina and Heyward (19)	91	18–73	SCI lesion level not reported	FFM = 18.874+ Ht^2^/R (0.367) + W (0.253) – age (0.081) – sex (5.384)	3.2	0.87
Buchholz et al. (33)	93 (19 males)	34.2 ± 8.8	Paraplegia	• FFM = TBW/0.732 • TBW = 2.11 – (0.1 x age) + (3.45 × sex) + (0.34 × W) + (28) Ht^2^/R – (0.086) sex. W	1.86	0.95

*FFM, Fat Free Mass in kilograms; TBW, Total Body Water in liters; sex is 0 for men and 1 for women; W, weight in kilograms; Ht, height (cm); R, resistance (ohms); SEE, standard error of the estimate; R^2^, determination coefficient*.

### Bioelectrical impedance vector analysis (BIVA)

BIVA tolerance consists of plotting the experimental data in a bivariate graph considering the 95th, 75th, and 50th vector percentiles of the Z-score of the reference population. Considering the plotting position of the experimental data, it is possible to suggest an interpretation: abnormal situation, when experimental data are positioned outside of the 95th percentile ellipsis; high body cell mass, when experimental data are located above the long axis of the ellipsis; hypohydration, when experimental data are positioned to the right of the short axis of the ellipsis. Total body water is inversely related to the length of the impedance vector, and a combination of the vector length and its direction is defined as phase angle (PhA) (27). The reference population for c-SCI used in the BIVA graph was obtained from the dataset of Sun et al. and Buccholz et al. predictive equations, with similar sex, age, and BMI range as the present study. R and Xc values from the Kocina et al. predictive equation were not available for BIVA use; for this reason, they were not applied in this study.

### Statistical analysis

Statistical analyses were performed using SPSS software version 19 (IBM Corporation, Armonk, NY, USA) and MedCalc Statistical Software version 14.8.1 (MedCalc Software, Ostend, Belgium; http://www.medcalc.org; 2014). Statistical tests were considered significant at the significance level of 5% (*P* ≤ 0.05).

Anthropometric measurements and body composition compartments were expressed as median and interquartile range. Mann-Whitney test was used to determine differences between groups, according to physical exercise (physically active and non-active). Wilcoxon test was used to determine the difference between the FFM values obtained by the three BIA predictive equations and values obtained using DXA. Lin's concordance correlation coefficient (35) was used considering the strength-of-agreement criteria described by (36) (almost perfect: >0.99; substantial: 0.95–0.99; moderate: 0.90–0.95; and poor: <0.90). The root means square error (RSME) between FFM observed (DXA) and predicted equation values were calculated. The mean absolute percentage error (MAPE) was calculated in percentage (37) for the FFM predictions performed with the two methods. BIVA was performed using specific software, which was kindly provided by Dr. Antonio Piccoli (*in memoriam*- Institute of Internal Medicine, Division of Nephrology, and Clinical Nutrition Unit, University of Padova, Padova, Italy).

## Results

General characteristics of the participants are shown in Table 2. The PA group showed lower chronological age (*p* = 0.029) and shorter time since injury (*p* = 0.023) than NPA. Since there was no significant difference between the groups in other variables, the statistical analysis considered all participants.

**Table 2:** c-SCI individuals' general characteristics according to physical exercise.

**Variable**	**Median (IQR), PA (*n* = 13)**	**Median (IQR), NPA (*n* = 10)**	***P*-value**
Age (years)	25.0 (23.0–33.0)	36.5 (32.8–40.0)	0.029
Time since injury (years)	3.3 (2.8–7.4)	14.0 (9.7–17.9)	0.023
Total body mass (kg)	64.9 (59.7–73.2)	70.9 (61.4–74.8)	0.619
Height (cm)	172.0 (165.0–179.0)	172.2 (169.3–176.1)	0.803
BMI (kg/m^2^)	21.9 (20.9–23.2)	23.4 (20.8–24.5)	0.784
**FFM (kg)**			
DXA	47.5 (45.2–50.1)	45.3 (41.4–48.4)	0.313
Sun et al. (34)	40.3 (38.8–47.7)	43.0 (37.5–46.7)	0.927
Kocina and Heyward (19)	52.9 (51.1–55.9)	52.5 (49.0–56.5)	0.642
Buchholz et al. (33)	46.7 (45.0–49.9)	46.0 (42.0–51.4)	0.522

*Values are presented as median and interquartile range [IQR (25% and 75% percentiles)]. BMI, body mass index; FFM, fat free mass; DXA, dual energy X-ray absorptiometry. Mann–Whitney test P <0.05 was considered significant*.

Comparison between FFM values obtained using DXA and BIA showed that Kocina and Heyward (*p* ≤ 0.001) and Sun et al. (*p* = 0.012) predictive equations were significantly different from DXA. However, Buchholz et al. predictive equation showed no difference in FFM when compared to the value obtained using DXA (Table 3).

**Table 3:** FFM obtained using DXA and BIA predictive equation (kg).

	**DXA**	**Sun et al. (34)**	**Kocina and Heyward (19)**	**Buchholz et al. (33)**
Median	47.0	41.7	52.9	46.7
IQR (25–75%)	43.8–49.3	38.1–47.5	50.3–56.3	43.5–51.0
*p*-value		0.012	<0.001	0.999

*Interquartile range (IQR). p-values by Wilcoxon test. P ≤ 0.05 was considered significant. MAPE, mean absolute percentage error*.

**Figure 1:**
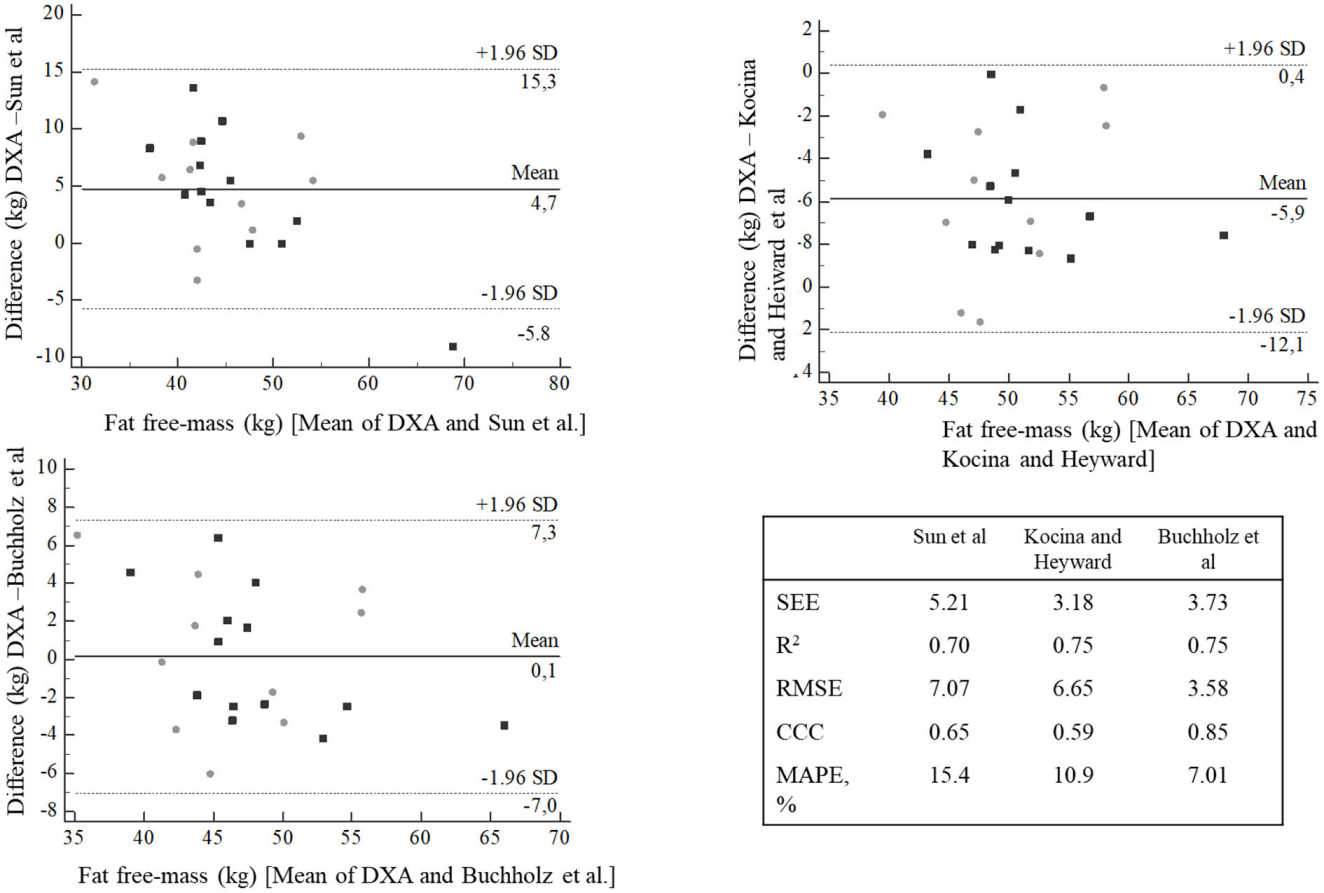
Bland-Altman plots agreement between fat free-mass predictive equations compared with DXA. Gray circle = NPA, black square = PA. SEE, Standard Error of the Estimate; *R*^2^, coefficient of determination; CCC, concordance correlation coefficient; MAPE, mean absolute percentage error.

Bland-Altman plots showed that Kocina and Heyward predictive equation presented higher bias (−5.9 kg) than Sun et al. (4.7 kg) and Buchholz et al. (0.1 kg) predictive equations. However, other concordance parameters showed that Sun et al. predictive equation presented higher SEE (5.21), RMSE (7.07) and MAPE (15.4%), and lower *R*^2^ (0.70) and CCC (0.65) than Kocina and Heyward, and Buchholz et al. predictive equations (Figure 1).

**Figure 2:**
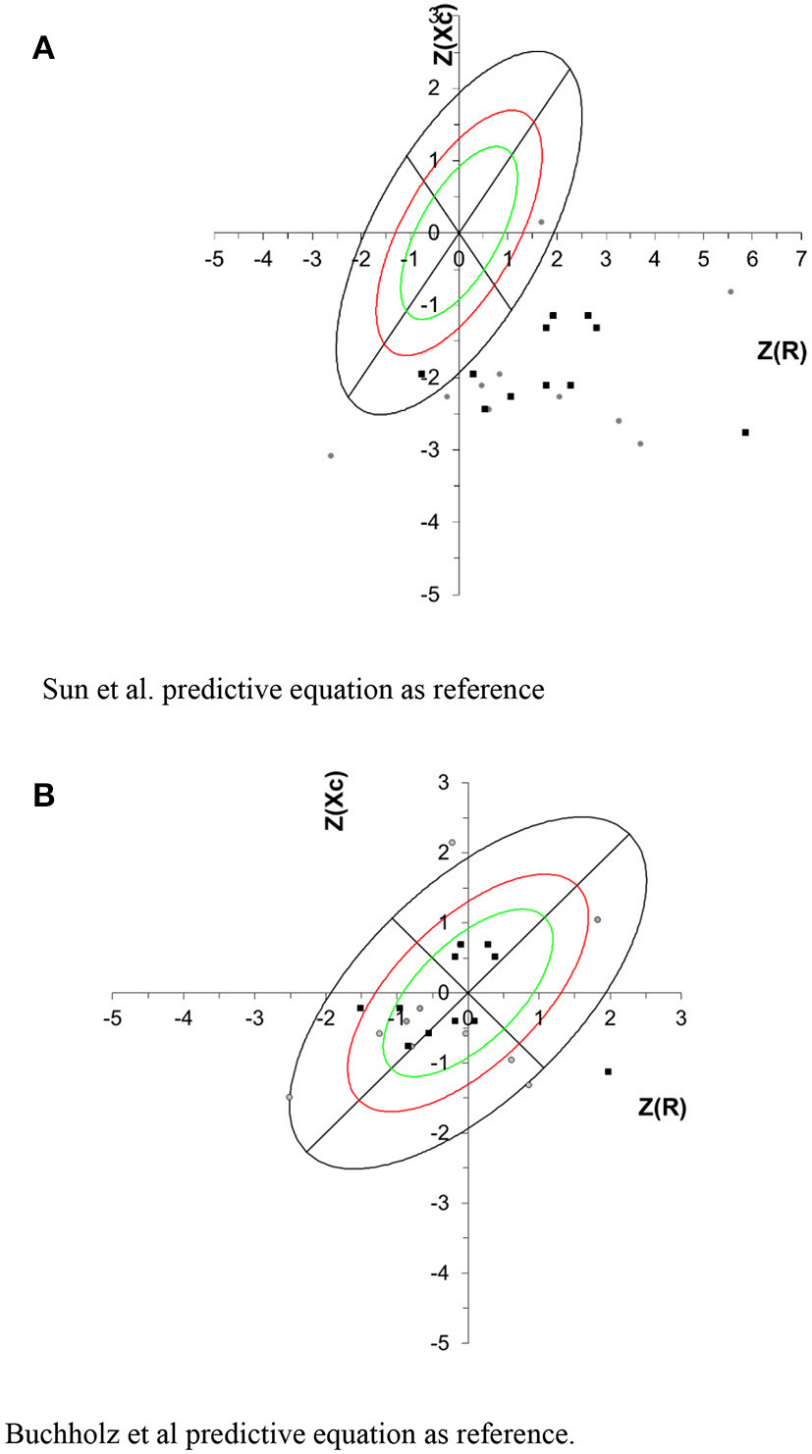
Tolerance intervals for the impedance vector are described as tolerance ellipses of 50% (green), 75% (red) and 95% (black) calculated from the healthy reference population. Gray circle represents the NPA group, and black square represents the PA group. For all participants the correlation between R/H and Xc/H was 0.363. **(A)** Sun et al. predictive equation as reference. **(B)** Buchholz et al. predictive equation as reference.

When Z-score Xc and R values obtained by Sun et al. were used as reference population in the BIVA graph, the vectors were displaced below the high axis of the tolerance ellipses, showing little body cell mass. However, when plotted with Z-score Xc and R values obtained by Buchholz et al. predictive equation, most individuals were located within the tolerance ellipses (Figure 2).

## Discussion

This pilot study showed that Buchholz et al. FFM-BIA predictive equation presented the highest concordance coefficient with FFM obtained using DXA; however, this is not sufficient to recommend it to c-SCI individuals. Therefore, BIVA could be used with caution, because according to the population reference used, different conclusions can be observed, such as demonstrated in this study. To our knowledge this is the first study to test BIVA in SCI individuals.

Regular physical activity practice for at least 150 min/week may be responsible for the improvement of body composition, hormonal profile and bone health in SCI individuals (38–41). Despite the benefits of exercise, this study failed to observe differences in the FFM values between the PA and NPA groups, possibly due to significant difference in age and time of injury between the groups.

Sun et al. predictive equation was developed with 734 able-bodied physically active males, including elderly individuals. c-SCI individuals have a body composition similar to that of the elderly population, presenting, for example, FFM loss and fat mass increase (42). In the present study, FFM obtained by Sun et al. predictive equation presented higher MAPE value (15.4%) than Kocina and Heyward and Buchholz et al. FFM predictive equations, and lower concordance coefficient than Buccholz et al. predictive equation. Possibly this happens because Sun's predictive equation was developed and validated for elderly individuals.

Plotting the R and Xc z-score in BIVA tolerance graph, using Sun's predictive equation as reference, it was observed that all c-SCI individuals were located under the highest axis, which can be attributed to the lowest body cell mass (BCM). BCM is the metabolically active cell mass involved in energy production and energy expenditure and is an important component of FFM (43). Our results using Sun's et al. predictive equation in BIVA were consistent with the c-SCI individuals body composition profile when compared to able bodied individuals.

Kocina and Heyward predictive equation was developed for SCI adults; however, the abstract does not present information about sample size, height and time of injury of the participants, resistance and reactance values. In the present study, FFM values obtained using the Kocina and Heyward predictive equation presented a high mean error percentage represented by MAPE (10.9%) and the lowest value of concordance coefficient, suggesting that despite having been developed with SCI individuals, it was not suitable for the group of the present study, possibly due to the wide range age group (18–73 years). The Kocina and Heyward predictive equation was not used in BIVA as a reference group, because there is no information on the bioelectrical data.

Buchholz et al. FFM BIA predictive equation was developed for healthy elderly subjects and tested and validated for paraplegic individuals, using the TBW method as reference, which reflects the lean soft tissue as muscle mass, considering the hydration constant (0.732) (44). In the present study, FFM values obtained using Buchholz et al. predictive equation presented higher concordance coefficient and lower MAPE than Kocina and Heyward and Sun et al. predictive equations. FFM values obtained by Panisset et al. using Buchholz et al. predictive equation observed a good concordance and low underestimation bias, in acute SCI obese individuals, whereas this study aimed for chronic c-SCI individuals.

In the present study, BIVA tolerance ellipses of SCI individuals shifted to the right, when Buchholz et al. predictive equation was used as reference, indicating high cell mass and fluid content, which can be attributed to better cell functioning (45). This profile is consistent when a specific equation for SCI individuals was used as reference; differently observed when the Sun et al. equation was used as a reference.

In conclusion, the present study was able to show that BIVA is sensitive to adaptations in body composition and to hydration status, responding consistently to the equations used as a reference, despite some limitations, such as: lack of hydration status control, and difference between age and time of physical exercise. In addition, future studies on new FFM predictive equations, specific for SCI individuals, should be developed according to spinal lesion characteristics and with a more representative sample size. The inaccuracies in assessing body composition and fluids at the c-SCI group may compromise an adequate assessment and monitoring of body fluids and may interfere with the clinical care of this specific and vulnerable group. Therefore, caution should be applied when interpreting data extracted from generalized equations.

## Data availability statement

The raw data supporting the conclusions of this article will be made available by the authors, without undue reservation.

## Ethics statement

The studies involving human participants were reviewed and approved by National Research Ethics System (COEP 052/2009). The patients/participants provided their written informed consent to participate in this study.

## Author contributions

FF performed data collection. AB, AC, FF, and JK analyzed the data and participated in the conceptualization of the work. AB wrote the original manuscript. AC and JK were involved in proofreading and editing. All authors read and agreed with the published version of the manuscript.

## Funding

This work was supported in part by Fundação Carlos Chagas Filho de Amparo à Pesquisa do Estado do Rio de Janeiro (E-26/111.778/2008, FAPERJ, Brazil). The Coordenação de Aperfeiçoamento de Pessoal de Nível Superior - Brazil (CAPES) sponsored a part of this study - Finance Code 001, and the PROPESP/PAPQ UFPA.

## Conflict of interest

Author FF was employed by Brazilian Paralympic Committee. The remaining authors declare that the research was conducted in the absence of any commercial or financial relationships that could be construed as a potential conflict of interest.

## Publisher's note

All claims expressed in this article are solely those of the authors and do not necessarily represent those of their affiliated organizations, or those of the publisher, the editors and the reviewers. Any product that may be evaluated in this article, or claim that may be made by its manufacturer, is not guaranteed or endorsed by the publisher.

## References

[1] Mneimneh F Moussalem C Ghaddar N Aboughali K Omeis I. Influence of cervical spinal cord injury on thermoregulatory and cardiovascular responses in the human body: literature review. J Clin Neurosci Off J Neurosurg Soc Aust. (2019) 69:7–14. 10.1016/j.jocn.2019.08.022 31447370

[2] Groah SL Nash MS Ward EA Libin A Mendez AJ Burns P. Cardiometabolic risk in community-dwelling persons with chronic spinal cord injury. J Cardiopulm Rehabil Prev. (2011) 31:73–80. 10.1097/HCR.0b013e3181f68aba 21045711

[3] Chilibeck PD Guertin PA. Locomotor training and factors associated with blood glucose regulation after spinal cord injury. Curr Pharm Des. (2017) 23:1834–44. 10.2174/1381612822666161216120546 27981906

[4] Chain A Koury JC Bezerra FF. Physical activity benefits bone density and bone-related hormones in adult men with cervical spinal cord injury. Eur J Appl Physiol. (2012) 112:3179–86. 10.1007/s00421-011-2303-7 22218778

[5] D'Oliveira GLC Figueiredo FA Passos MCF Chain A Bezerra FF Koury JC. Physical exercise is associated with better fat mass distribution and lower insulin resistance in spinal cord injured individuals. J Spinal Cord Med. (2014) 37:79–84. 10.1179/2045772313Y.0000000147 24090139 PMC4066554

[6] Koury JC Passos MCF Figueiredo FA Chain A Franco JG. Time of physical exercise practice after injury in cervical spinal cord-injured men is related to the increase in insulin sensitivity. Spinal Cord. (2013) 51:116–119. 10.1038/sc.2012.85 22777489

[7] Eldahan KC Rabchevsky AG. Autonomic dysreflexia after spinal cord injury: systemic pathophysiology and methods of management. Auton Neurosci Basic Clin. (2018) 209:59–70. 10.1016/j.autneu.2017.05.002 28506502 PMC5677594

[8] Popa C Popa F Grigorean VT Onose G Sandu AM Popescu M. Vascular dysfunctions following spinal cord injury. J Med Life. (2010) 3:275–85. 20945818 PMC3019008

[9] Liberman K Forti LN Beyer I Bautmans I. The effects of exercise on muscle strength, body composition, physical functioning and the inflammatory profile of older adults: a systematic review. Curr Opin Clin Nutr Metab Care. (2017) 20:30–53. 10.1097/MCO.0000000000000335 27755209

[10] Sardinha LB Correia IR Magalhães JP Júdice PB Silva AM Hetherington-Rauth M. Development and validation of BIA prediction equations of upper and lower limb lean soft tissue in athletes. Eur J Clin Nutr. (2020) 74:1646–52. 10.1038/s41430-020-0666-8 32472025

[11] Sedlmeier AM Baumeister SE Weber A Fischer B Thorand B Ittermann T. Relation of body fat mass and fat-free mass to total mortality: results from 7 prospective cohort studies. Am J Clin Nutr. (2021) 113:639–46. 10.1093/ajcn/nqaa339 33437985

[12] Shepherd JA Ng BK Sommer MJ Heymsfield SB. Body composition by DXA. Bone. (2017) 104:101–5. 10.1016/j.bone.2017.06.010 28625918 PMC5659281

[13] Martin Ginis KA van der Scheer JW Latimer-Cheung AE Barrow A Bourne C Carruthers P. Evidence-based scientific exercise guidelines for adults with spinal cord injury: an update and a new guideline. Spinal Cord. (2018) 56:308–21. 10.1038/s41393-017-0017-3 29070812

[14] Karlsson A-K. Autonomic dysfunction in spinal cord injury: clinical presentation of symptoms and signs. Prog Brain Res. (2006) 152:1–8. 10.1016/S0079-6123(05)52034-X 16198689

[15] Ma Y de Groot S Weijs PJM Achterberg W Adriaansen J Janssen TWJ. Accuracy of bioelectrical impedance analysis and skinfold thickness in the assessment of body composition in people with chronic spinal cord injury. Spinal Cord. (2022) 60:228–36. 10.1038/s41393-021-00682-w 34385607

[16] Mojtahedi MC Valentine RJ Evans EM. Body composition assessment in athletes with spinal cord injury: comparison of field methods with dual-energy x-ray absorptiometry. Spinal Cord. (2009) 47:698–704. 10.1038/sc.2009.20 19290014

[17] Desport JC Preux PM Guinvarc'h S Rousset P Salle JY Daviet JC. Total body water and percentage fat mass measurements using bioelectrical impedance analysis and anthropometry in spinal cord-injured patients. Clin Nutr Edinb Scotl. (2000) 19:185–190. 10.1054/clnu.1999.0122 10895109

[18] Kyle UG Bosaeus I De Lorenzo AD Deurenberg P Elia M Manuel Gómez J. Bioelectrical impedance analysis-part ii: utilization in clinical practice. Clin Nutr Edinb Scotl. (2004) 23:1430–53. 10.1016/j.clnu.2004.09.012 15556267

[19] Kocina PS Heyward VH. Validation of a bioimpedance equation for estimating fat-free mass of spinal cord injured adults. Med Sci Sports Exerc. (1997) 29:55.

[20] Panisset MG Desneves K Ward LC Rafferty J Rodi H Roff G. Bedside quantification of fat-free mass in acute spinal cord injury using bioelectrical impedance analysis: a psychometric study. Spinal Cord. (2018) 56:355–65. 10.1038/s41393-017-0035-1 29284797

[21] Desneves KJ Panisset MG Galea MP Kiss N Daly RM Ward LC. Comparison of segmental lean tissue mass in individuals with spinal cord injury measured by dual energy x-ray absorptiometry and predicted by bioimpedance spectroscopy. Spinal Cord. (2021) 59:730–37. 10.1038/s41393-020-00568-3 33077901

[22] Yamada Y Watanabe Y Ikenaga M Yokoyama K Yoshida T Morimoto T. Comparison of single- or multifrequency bioelectrical impedance analysis and spectroscopy for assessment of appendicular skeletal muscle in the elderly. J Appl Physiol Bethesda Md 1985. (2013) 115:812–8. 10.1152/japplphysiol.00010.2013 23813532

[23] Tran AP Warren PM Silver J. The biology of regeneration failure and success after spinal cord injury. Physiol Rev. (2018) 98:881–917. 10.1152/physrev.00017.2017 29513146 PMC5966716

[24] Siri WE. Composition from fluid spaces AnBody d density: analysis of methods. 1961. Nutr Burbank Los Angel Cty Calif. (1993) 9:480–91. Discussion 480, 492. 8286893

[25] Piccoli A. Patterns of bioelectrical impedance vector analysis: learning from electrocardiography and forgetting electric circuit models. Nutr Burbank Los Angel Cty Calif. (2002) 18:520–1. 10.1016/S0899-9007(02)00771-2 12044826

[26] Piccoli A Rossi B Pillon L Bucciante G. A new method for monitoring body fluid variation by bioimpedance analysis: the RXc graph. Kidney Int. (1994) 46:534–9. 10.1038/ki.1994.305 7967368

[27] Piccoli A Nigrelli S Caberlotto A Bottazzo S Rossi B Pillon L. Bivariate normal values of the bioelectrical impedance vector in adult and elderly populations. Am J Clin Nutr. (1995) 61:269–70. 10.1093/ajcn/61.2.269 7840061

[28] Piccoli A. Bioelectric impedance measurement for fluid status assessment. Contrib Nephrol. (2010) 164:143–52. 10.1159/000313727 20428000

[29] Strapazzon G Pun M Cappello TD Procter E Lochner P Brugger H. Total body water dynamics estimated with bioelectrical impedance vector analysis and B-type natriuretic peptide after exposure to hypobaric hypoxia: a field study. High Alt Med Biol. (2017) 18:384–91. 10.1089/ham.2017.0056 29035594 PMC5743030

[30] Thomas DT Erdman KA Burke LM. American college of sports medicine joint position statement. Nutrition and athletic performance. Med Sci Sports Exerc. (2016) 48:543–68. 10.1249/MSS.0000000000000852 26891166

[31] Bauermann A de Sá KSG Santos ZA Costa E Silva AA. Supplementation and performance for wheelchair athletes: a systematic review. Adapt Phys Act Q APAQ. (2021) 39:1–15. 10.1123/apaq.2020-0241 34758458

[32] Crabtree NJ Arabi A Bachrach LK Fewtrell M El-Hajj Fuleihan G Kecskemethy HH. International society for clinical densitometry dual-energy x-ray absorptiometry interpretation and reporting in children and adolescents: the revised 2013 ISCD pediatric official positions. J Clin Densitom Off J Int Soc Clin Densitom. (2014) 17:225–42. 10.1016/j.jocd.2014.01.003 24690232

[33] Buchholz AC McGillivray CF Pencharz PB. The use of bioelectric impedance analysis to measure fluid compartments in subjects with chronic paraplegia. Arch Phys Med Rehabil. (2003) 84:854–61. 10.1016/S0003-9993(02)04950-X 12808538

[34] Sun SS Chumlea WC Heymsfield SB Lukaski HC Schoeller D Friedl K. Development of bioelectrical impedance analysis prediction equations for body composition with the use of a multicomponent model for use in epidemiologic surveys. Am J Clin Nutr. (2003) 77:331–40. 10.1093/ajcn/77.2.331 12540391

[35] Lin LI. A concordance correlation coefficient to evaluate reproducibility. Biometrics. (1989) 45:255–68. 10.2307/2532051 2720055

[36] McBride G. A proposal for strength-of-agreement criteria for lin's concordance correlation coefficient. NIWA Client Rep HAM. (2005) 45:307–10.

[37] Chen C Twycross J Garibaldi JM. A new accuracy measure based on bounded relative error for time series forecasting. PLoS ONE. (2017) 12:e0174202. 10.1371/journal.pone.0174202 28339480 PMC5365136

[38] Castro MJ Apple DF Hillegass EA Dudley GA. Influence of complete spinal cord injury on skeletal muscle cross-sectional area within the first 6 months of injury. Eur J Appl Physiol. (1999) 80:373–8. 10.1007/s004210050606 10483809

[39] Gater DR. Obesity after spinal cord injury. Phys Med Rehabil Clin N Am. (2007) 18:333–51, vii. 10.1016/j.pmr.2007.03.004 17543776

[40] Gordon PS Farkas GJ Gater DR. Neurogenic obesity-induced insulin resistance and type 2 diabetes mellitus in chronic spinal cord injury. Top Spinal Cord Inj Rehabil. (2021) 27:36–56. 10.46292/sci20-00063 33814882 PMC7983643

[41] Gorla JI Costa e Silva AA Borges M Tanhoffer RA Godoy PS Calegari DR. Impact of wheelchair rugby on body composition of subjects with tetraplegia: a pilot study. Arch Phys Med Rehabil. (2016) 97:92–6. 10.1016/j.apmr.2015.09.007 26433046

[42] St-Onge M-P Gallagher D. Body composition changes with aging: the cause or the result of alterations in metabolic rate and macronutrient oxidation? Nutr Burbank Los Angel Cty Calif. (2010) 26:152–5. 10.1016/j.nut.2009.07.004 20004080 PMC2880224

[43] Wells JCK Murphy AJ Buntain HM Greer RM Cleghorn GJ Davies PSW. Adjusting body cell mass for size in women of differing nutritional status. Am J Clin Nutr. (2004) 80:333–6. 10.1093/ajcn/80.2.333 15277153

[44] Pialoux V Mischler I Mounier R Gachon P Ritz P Coudert J. Effect of equilibrated hydration changes on total body water estimates by bioelectrical impedance analysis. Br J Nutr. (2004) 91:153–9. 10.1079/BJN20031031 14748949

[45] Xiao J Purcell SA Prado CM Gonzalez MC. Fat mass to fat-free mass ratio reference values from NHANES III using bioelectrical impedance analysis. Clin Nutr Edinb Scotl. (2018) 37:2284–7. 10.1016/j.clnu.2017.09.021 29056283

